# Improved poliovirus d-antigen yields by application of different Vero cell cultivation methods

**DOI:** 10.1016/j.vaccine.2014.02.022

**Published:** 2014-05-19

**Authors:** Yvonne E. Thomassen, Olaf Rubingh, René H. Wijffels, Leo A. van der Pol, Wilfried A.M. Bakker

**Affiliations:** a Institute for Translational Vaccinology, Process Development, PO BOX 450, Bilthoven 3720 AL, The Netherlands; b Wageningen University, Bioprocess Engineering, PO BOX 8129, Wageningen 6700 EV, The Netherlands

**Keywords:** Microcarriers, Perfusion, Feed, Recirculation, Batch, Adherent

## Abstract

Vero cells were grown adherent to microcarriers (Cytodex 1; 3 g L^−1^) using animal component free media in stirred-tank type bioreactors. Different strategies for media refreshment, daily media replacement (semi-batch), continuous media replacement (perfusion) and recirculation of media, were compared with batch cultivation. Cell densities increased using a feed strategy from 1 × 10^6^ cells mL^−1^ during batch cultivation to 1.8, 2.7 and 5.0 × 10^6^ cells mL^−1^ during semi-batch, perfusion and recirculation, respectively.

The effects of these different cell culture strategies on subsequent poliovirus production were investigated. Increased cell densities allowed up to 3 times higher d-antigen levels when compared with that obtained from batch-wise Vero cell culture. However, the cell specific d-antigen production was lower when cells were infected at higher cell densities. This cell density effect is in good agreement with observations for different cell lines and virus types. From the evaluated alternative culture methods, application of a semi-batch mode of operations allowed the highest cell specific d-antigen production.

The increased product yields that can easily be reached using these higher cell density cultivation methods, showed the possibility for better use of bioreactor capacity for the manufacturing of polio vaccines to ultimately reduce vaccine cost per dose. Further, the use of animal-component-free cell- and virus culture media shows opportunities for modernization of human viral vaccine manufacturing.

## Highlights

• Vero cells were grown in batch, semi-batch, perfusion and recirculation strategies.
• At high cell densities (to 5 × 10^6^ cells mL^−1^) cells were infected with poliovirus.
• Increased cell densities allowed 3 fold increase in d-antigen yield.
• Cell specific d-antigen yields were lower at higher cell densities.
• The semi-batch cultivation strategy is most promising for optimization.

## 1 Introduction

Recently, we have produced Sabin-IPV (inactivated polio vaccine based on attenuated Sabin strains) clinical lots under cGMP for phase I safety (and indicative immunogenicity) studies in human adults and infants [1], [2]. The applied production process was based on a scale-down model of the Salk-IPV manufacturing process [3]. The use of this scale-down model allowed fast development of a first generation Sabin-IPV, for which the specifications are closely related to that for the regular IPV product [2]. Parallel to this fast-track development an optimization and modernization research program for the manufacturing of Sabin-IPV was started. Examples of modernization are replacement of the used animal derived components (e.g. bovine serum and porcine trypsin) and antibiotics. These components should preferably be omitted (for the antibiotics primarily to prevent any potential allergic reaction), or respectively replaced by animal component free (ACF) alternatives to minimize the risk of adverse effects (e.g. the potential transfer of viruses and/or prions). Moreover, a better scientific understanding of the process, resulting in improved process control and ability for troubleshooting, can be created. Optimization improvements can possibly be found in the currently used, low cell densities (1 × 10^6^ cells mL^−1^). Assuming comparable virus quality and yields per cell, the use of increased cell densities can potentially result in more efficient use of bioreactor capacity, and ultimately reduce the cost per dose. The demand for IPV is increasing as in 2012 the WHO SAGE group advised all countries to introduce at least one dose IPV in their routine immunization schedules [4]. With the increased IPV demands, which will further increase after oral polio vaccine (OPV) cessation, the production capacity will have to increase by scale-up and optimization causing the current IPV price of $ 3.00 per dose to decrease to $ 0.52–$ 1.95 [5]. This is still four to fifteen times the current price of OPV (cost per dose $ 0.14), the vaccine used in most countries. Process optimization for IPV manufacturing will be needed to be able to further reduce manufacturing costs below $ 0.50 to keep polio vaccination economically feasible when switching from OPV to IPV [6].

Here we report initial studies where four different adherent Vero cell cultivation methods were applied using ACF cell culture media: (i) batch, the currently used method for Sabin-IPV preparation; (ii) semi-batch, where daily media refreshments were applied; (iii) perfusion where continuous media refreshment was applied; and (iv) recirculation where media was circulated through the bioreactor and re-used. With these commonly known cell culture methods [7] higher cell densities were obtained and the subsequent virus culture, also using ACF virus culture media, produced higher quantities of infectious and immunogenic poliovirus.

## 2 Materials and methods

### 2.1 Cells, virus

Vero cells obtained from WHO (10-87) originally derived from ATCC (CCL-81) were used as host for poliovirus production. Poliovirus seeds [1] Sabin type 1 (LSc 2ab KP_2_; SO + 3), Sabin type 2 (P712 Ch2ab-KP_2_; SO + 3) and Sabin type 3 (Lot 457-III-Pfizer; RSO3) were used.

### 2.2 Cultivation methods

#### 2.2.1 Pre-cultures

Vero cells were cultured in T-flasks and Hyperflasks (Corning) in VP-SFM (Invitrogen) to expand the cell number. After trypisinization (TrypLE Select; Invitrogen) cells were resuspended in VP-SFM and added to the bioreactor.

#### 2.2.2 Bioreactor Vero cell cultures

Different cultivation methods have been applied where Vero cells were grown adherent to microcarriers (3 g L^−1^ Cytodex 1; GE Healthcare). The cultures were maintained at pH 7.2, 37 °C, 50% dissolved oxygen (DO) by headspace aeration only (1 L min^−1^) and sampled at least once a day.

Cell cultures were carried out in standard glass stirred-tank type bioreactors, optionally equipped with a spin filter (70 μm) to retain cells on microcarriers in the bioreactor when needed (perfusion and recirculation culture mode). Alternatively, a harvest pipe with a 75 μm sieve was used to remove media while retaining microcarriers. Cultivations were controlled using Sartorius DCU-3 control units and MFCS-win software (Sartorius AG, Melsungen, Germany).

Batch cultivations were carried out at 4 L working volume with inoculation densities of 0.1 × 10^6^ cells mL^−1^. During cultivation, glucose and glutamine were added by bolus feeding to 10 mM glucose and 2 mM glutamine when concentrations were below 5 mM and 0.5 mM respectively.

Semi-batch cultivations were essentially performed as described by Mendonça (1998) [8] at 3 L working volume with an inoculation density of 0.1 × 10^6^ cells mL^−1^. From day two onwards, daily 1 L culture medium (1/3 culture volume) was replaced with fresh medium. Media replacement was done after sedimentation of the microcarriers without agitation. In addition, bolus feeding of glucose and glutamine was done once 4 days after the start of cultivation to obtain concentrations of 20 mM glucose and 2 mM glutamine.

Perfusion cultivations were carried out using 1.5 L working volume. Cells were inoculated at 0.1 × 10^6^ cells mL^−1^ and retained in the bioreactor. After 2 days of batch cultivation, continuous media feed was started at 1.5 L day^−1^ (1 culture volume per day). Media feed rate was kept constant for the remainder of the perfusion cultures.

Recirculation cultures, where cells are retained in the bioreactor (3 L working volume) while medium (15 L total volume = culture volume + circulated volume) is circulated, were carried out essentially as described previously [9]. Cells were inoculated at a cell density of 0.6 × 10^6^ cells mL^−1^. After 1 day, a continuous recirculation flow was started with 15 L day^−1^ (5 culture volumes per day), followed by daily increments to 22 L day^−1^ and 30 L day^−1^ (respectively 7.3 and 10 culture volume per day) at days 3 and 4.

#### 2.2.3 Virus culture

Prior to virus infection, using the same bioreactor vessel used for Vero cell culture, the media feed was stopped and pH, DO and temperature settings were adjusted to 7.4, 25% and 32.5 °C, respectively. Media was not refreshed but glucose and glutamine were fed when concentrations were below 5 mM and 0.5 mM, respectively. Cells were infected with poliovirus with an MOI (multiplicity of infection) of 0.01. Virus cultivation was considered finished when 100% CPE (cytopathic effect) was observed microscopically.

### 2.3 Analytics

Cells were counted daily using a Nucleocounter NC-100 (Chemometec).

Cell culture metabolites such as glucose, lactate, glutamine, glutamate and ammonia were monitored using a Bioprofile 100 Plus (Nova Biomedical Waltham, MA).

Poliovirus was quantified with a virus titer assay as described previously [10]. The amount of d-antigen was assessed using a d-antigen ELISA [11].

## 3 Results

### 3.1 Vero cell growth

**Fig. 1:**
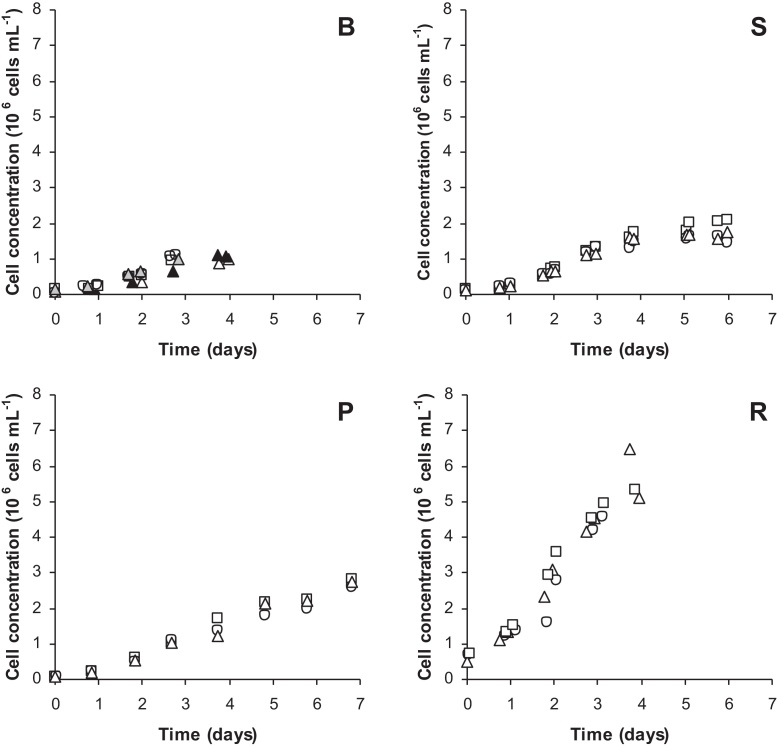
Growth curves of Vero cells using different cultivation modes batch (B), semi-batch (S), perfusion (P) and recirculation (R). Different batches are indicated with different grey shades and symbols, where squares, circles and triangles represent cultures that were infected with poliovirus type 1, 2 and 3 respectively. Infection of cultures was done at the end of the shown growth curve.

**Fig. 2:**
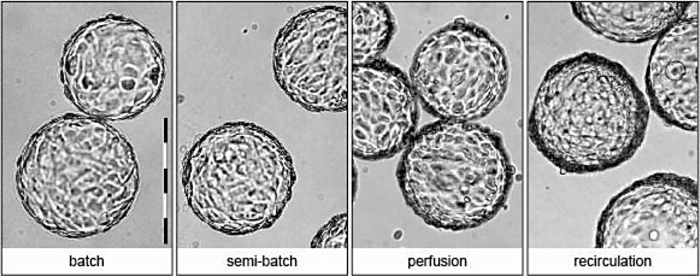
Photographs of Vero cells on microcarriers at the time of virus inoculation. Presence of multilayers at higher cell concentrations (1.0, 1.8, 2.7 and 5.0 × 10^6^ cells mL^−1^ for respectively batch, semi-batch, perfusion and recirculation) is clearly visible.

Vero cell cultures were performed in four different cultivation modes, batch, semi-batch, perfusion and recirculation. Batch cultivations were performed to obtain a reference growth curve for later comparison with the more sophisticated culture methods where either media is refreshed (semi-batch and perfusion) or circulated (recirculation). After 3–4 days of cultivation, a cell density at 1.0 × 10^6^ cells mL^−1^ was reached in batch cultivation with an average growth rate of 0.036 h^−1^ during exponential growth and a growth rate of 0.022 h^−1^ at the moment of virus infection on day 4 (Fig. 1; Table 1). At this point cells are present as a monolayer on the microcarriers (Fig. 2). Applying a daily partial medium renewal in a semi-batch mode allowed cell growth to continue and after 2 additional days of culture (6 days in total) a cell density of 1.8 × 10^6^ cells mL^−1^ was obtained. Here comparable growth rates to batch cultivation were observed. The growth rate declined during the feed phase from 0.034 h^−1^ at day 3 to 0.006 h^−1^ at day 6. Using a perfusion mode, where medium renewal is continuous, cell growth could be prolonged to yield a cell density of 2.7 × 10^6^ cells mL^−1^ in 7 days. The growth rates of the Vero cells were lower during the feed phase compared to the growth rates observed in semi-batch cultivations and decreased from 0.018 h^−1^ at day 3 to 0.005 h^−1^ at day 7. Cells were present in a multilayer on the microcarriers at these cell concentrations (Fig. 2). In the so-called recirculation method [9] cells were retained in the bioreactor while medium from an external container was circulated. When starting with an inoculation density of 0.6 × 10^6^ cells mL^−1^ a monolayer was already formed after one day of cultivation, and cells started to grow in a multilayer rapidly. Cell concentrations of 5.0 × 10^6^ cells mL^−1^ were found after a culture time of 4 days, while growth rates decreased linearly during the feed phase from 0.025 h^−1^ at day 2 to 0.0004 h^−1^ at day 4. It should be noted, that the latter cultivation method was applied using higher inoculation densities, which implies that a more extensive (or longer) pre-culture was needed.

### 3.2 Glucose and glutamine concentrations during cell culture

**Fig. 3:**
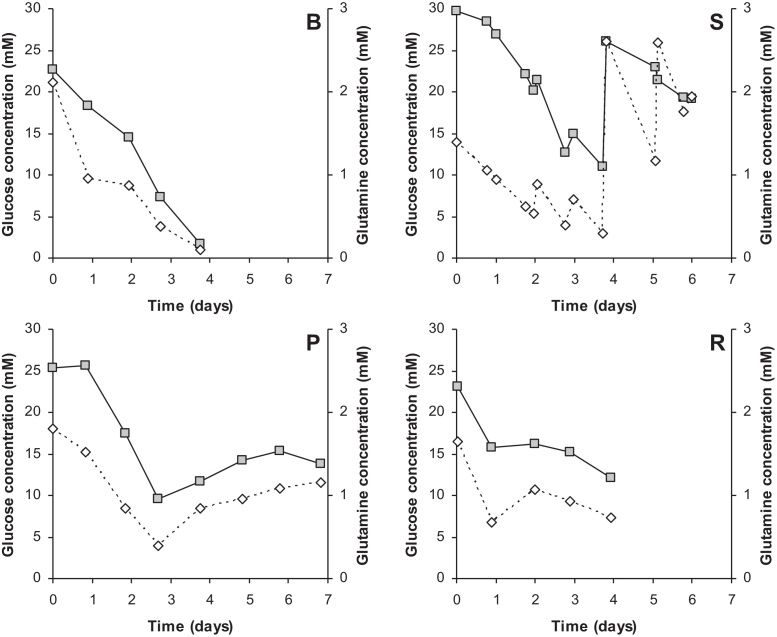
Glucose (squares) and glutamine (diamonds) concentrations present in Vero cell cultivations using batch (B), semi-batch (S), perfusion (P) or recirculation (R). Figures are averaged numbers. Daily media exchange was started at day 2 (S), media feed at day 3 (P) and circulation at day 1 (R).

The concentrations of glucose and glutamine were analyzed during the Vero cell growth in different cultivation modes. Glucose and glutamine concentrations decreased rapidly when the culture was in batch mode (Fig. 3). When media was refreshed daily (semi-batch) or continuously (perfusion) or when media was circulated (recirculation), sufficient glucose and glutamine were present during the complete cultivation time. During perfusion and recirculation cultivations it is clear that from the moment the feed was started the glucose and glutamine levels remained reasonably constant, whereas during semi-batch cultivations glucose and glutamine concentrations varied more. This was directly correlated to the feeding times. It should be noted that during semi-batch cultivations, an additional bolus feed of glucose and glutamine was given at day 4 (Fig. 3).

### 3.3 Lactate and ammonia concentrations

**Fig. 4:**
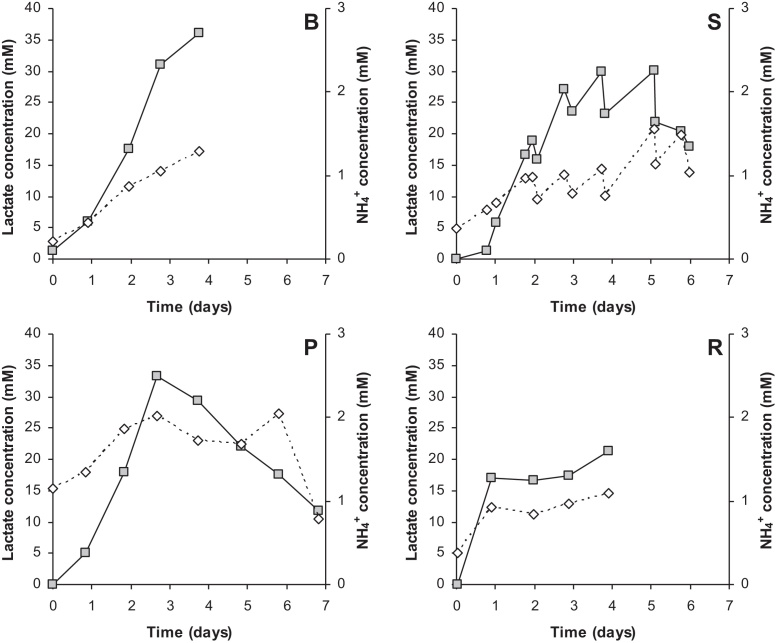
Lactate (squares) and NH_4_^+^ (diamonds) concentrations present in Vero cell cultivations using batch (B), semi-batch (S), perfusion (P) or recirculation (R). Figures are averaged numbers. Daily media exchange was started at day 2 (S), media feed at day 3 (P) and circulation at day 1 (R).

During the batch cultivation lactate and ammonia concentrations increased and within 3 days concentrations up to 30 mM lactate were reached. Daily media replacements allowed to keep lactate concentration below 30 mM whereas continuous media replacement lowered the lactate concentration. Recirculation of media caused a relative constant lactate and ammonia concentration during the cultivation time. Although lactate levels reach high concentrations (above 20 mM), the Vero cell growth continued and therefore it was concluded that this did not inhibit cell growth severely. Ammonia concentrations were below 2 mM under all growth conditions (Fig. 4).

### 3.4 Virus culture

To determine the variability in poliovirus yields, three cell cultures (in batch mode) were infected with poliovirus type 3. When virus culture was complete, virus titers were measured to determine the amount of infectious poliovirus and d-antigen was measured to quantify the amount of immunogenic poliovirus. The RSD (relative standard deviations) were 9% for the virus titer and 8% for the d-antigen concentration. Both are within 10%, which can be considered comparable. This means that cultures were very comparable as the virus titer assay is valid within 0.5 log (=6%) and the RSD for test reproducibility for the d-antigen ELISA is 10.6% [11]. Based on good virus culture reproducibility, it was chosen to compare the effects of different cell culture strategies on the virus yield with *n* = 1 for all three virus types.

**Fig. 5:**
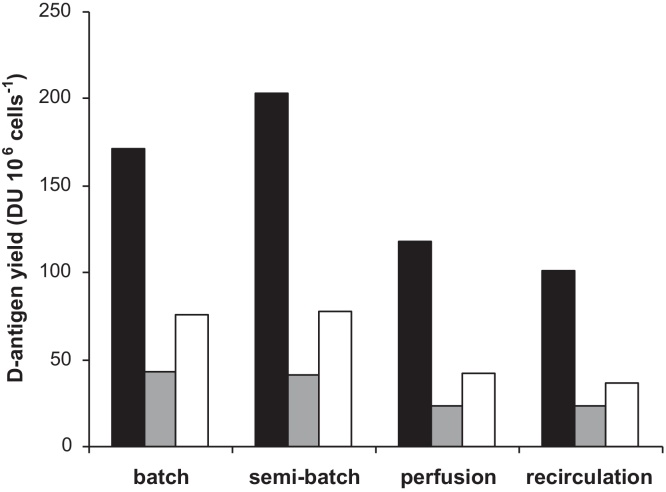
Cell specific d-antigen yields (poliovirus type 1, black; type 2, grey; type 3, white) after different cell culture methods. Similar virus culture conditions were applied.

Comparable virus titers were found independent of the cell culture method that was applied (Table 2). On the other hand, for all three poliovirus types differences in d-antigen concentrations were more pronounced. In all cases where media refreshments were used during cell cultures an increase of the d-antigen yield was observed, when compared with batch-wise cell culture. These increases ranged from approx. 1.5- to 2-fold when cell cultures were carried out in semi-batch and perfusion mode to approx. 2.4- to 2.9-fold when cell cultures were executed in recirculation mode. Since cell concentrations at the start of virus culture were different in the different settings (Table 1), the cell specific d-antigen yields were calculated and compared (Fig. 5). Cell specific d-antigen yields were the highest when virus culture was carried out based on semi-batch cell cultures for poliovirus type 1 and batch or semi-batch cell cultures for type 2 and 3. When perfusion or recirculation cultures were used prior to virus culture, the cell specific d-antigen yields were a factor 2 lower.

## 4 Discussion

The Vero cell line is one of the commonly used cell lines to produce viral vaccines [12]. Classic cell culture processes used in vaccine manufacturing are often based on batch-wise cell and virus cultivations followed by extensive downstream processing, concentration, purification and inactivation to yield a product [13], [14]. While downstream processing is important, the virus of interest is generated during upstream processing, i.e. cell and virus culture. It is also at this stage where the intrinsic product quality is determined. Whereas product yields may be related to both the cell concentration and the metabolic state of the cells, product quality is likely largely influenced by the cells metabolic condition and the virus culture conditions. In other words, the cell culture method may impact product quality. The cell cultures are discussed first, followed by the observed d-antigen levels as indicator of product quality.

The application of different cell culture strategies resulted in higher cell densities, up to 5 × 10^6^ cells mL^−1^ during recirculation cultures. These cell concentrations were at comparable levels to those previously reported for recirculation cultures [15]. In addition, the cell densities reached using perfusion, semi-batch and batch cultures were comparable to those reported by others [8], [16], [17].

At the higher cell densities, cells were growing in multilayers on the microcarriers. Recently it has been reported that the tumorgenicity of Vero cells is dependent not only on the passage level as reported previously [18], but also on the culture conditions [19]. The growth in dense cultures as well as the adaptation to serum free media may result in the acquisition of a tumorgenic phenotype. Moreover differences in cell morphology, i.e. the compactness of the monolayer, have been reported for Vero cell growth in different serum free media [20]. As such, tumorgenicity of the Vero cells growing in multilayers in a specific ACF medium should be investigated before these cells are used to produce clinical materials.

During all cell cultures, sufficient concentrations of glucose and glutamine were present. At the end of cell culture lactate concentrations were high, up to 36 mM during batch, approx. 20 mM during semi-batch and recirculation and 12 mM during perfusion cultures. Reports of high lactate concentrations during Vero cell growth are abundant when the carbon source is glucose, see for example Petiot et al. (2010) [17], and are caused by the overflow metabolism. High lactate concentrations may be prevented by using other carbon sources like fructose or galactose [8], [17]. The ammonia concentration was around 1 mM at the end of the cultivations, which is at an acceptable level that does not inhibit cell growth [21]. Since media was not changed prior to virus culture, these lactate and ammonia concentrations were present at virus infection.

The use of VP-SFM during cell and virus culture appeared beneficial for virus yields when compared to cultivation using serum containing medium during cell culture and M199 during virus culture. In earlier studies [1], using the latter media, d-antigen levels reported for production at 350-L scale were 120, 25 and 56 DU mL^−1^ for respectively Sabin poliovirus type 1, 2 and 3. The use of VP-SFM resulted in a 1.5 times higher level of antigenic product concentration using batch cultivations and 4 fold when using a recirculation culture prior to virus infection. It should be noted that here virus cultures were carried out using spent media. Regarding the nutrient and waste metabolite concentrations it might be even more beneficial to change the media prior to virus culture or to feed possible depleted nutrients during virus culture. This type of optimization may result in a favourable host cell metabolic condition with respect to virus production.

Differences in d-antigen yield per cell between batch or semi-batch and perfusion or recirculation were observed (Fig. 5). At higher cell densities the virus yield per cell decreased. This might be an example of the so-called “cell density effect” first described by Wood et al. [22] and observed for different virus cultivation systems [16], [20], [23], [24]. In several cases nutrient limitation or the presence of inhibitory factors may have caused this effect [16], [23], [24]. In others, the cause remains to be found [20], [25]. Here, the concentrations of the main nutrients, glucose and glutamine, and waste products, lactate and ammonia, were at less favourable levels during batch or semi-batch, while the highest specific product yields were observed under these conditions. We thus concluded that these concentrations are less relevant when compared with other phenomena that influence the cells ability to produce virus. These other aspects could be the growth rate at virus infection, the presence of multilayers, or the capacity (surface space) to continue growth after virus infection. Cell growth rates at time of virus infection were lower under all high cell density strategies compared to the growth rates observed in batch cultivations and thus do not explain for the difference in cell specific d-antigen yield observed between semi-batch and perfusion or recirculation cultures. Possibly, the presence of a multilayer has a more important negative effect. It could be that in a multilayer not all cells present on the microcarrier support virus replication in the same way. Moreover, the capacity to continue cell growth at the moment of virus infection may be important as the applied MOI was 0.01 which means 99% of the cells will not be infected during the first virus replication cycle and can potentially grow further. These topics are currently under investigation to be able to further optimize the virus culture at increased cell densities.

The highest virus yields, based on d-antigen concentrations, were observed using the recirculation mode for cell culture. At the first glance, to maximize bioreactor capacity, this seems to be the best choice. However, it should be mentioned that a larger pre-culture needs to be prepared as here the cell culture is started at 0.6 × 10^6^ instead of 0.1 × 10^6^ cells mL^−1^ used for the other cell culture strategies. Hence, extending the overall process throughput time. Further, considering the cell specific d-antigen productivity, the semi-batch cell culture strategy appeared to be a good alternative. In addition, this method can be applied in existing manufacturing equipment without large investments. At present, we are optimizing this method with respect to microcarrier concentration, feed frequency and feed/bioreactor volume ratio. In addition, adaptation of downstream processing to concentrate and purify the poliovirus obtained from increased cell density cultures is studied. Focus points are the filter load with cell debris during clarification and concentration and the removal of the increased concentrations of host cell proteins and host cell DNA during column chromatography. Also, product quality and immunogenicity after purification remains to be assessed. In that way, discrimination between intact virus particles and virus progeny, which may have attributed to the observed increased d-antigen levels, can be made.

## 5 Conclusions

This study shows that adherent Vero cell culture using different methods of medium refreshment allows higher cell densities. Increased cell densities allowed up to 3 times higher d-antigen levels when compared with that obtained from batch-wise Vero cell culture. The cell specific d-antigen production was lower when cells were infected at higher cell densities. Application of a semi-batch mode of operations allowed the highest cell specific d-antigen production, while 2 fold lower cell specific d-antigen yields were found using perfusion or recirculation cultures. This reduction may be related to the presence of multilayers of cells on the microcarriers, which were observed at higher cell densities that were reached using perfusion or recirculation mode. In our view, the most promising concept for polio d-antigen yield optimization would be semi-batch cultivations. This strategy has potential to be further improved and can be implemented in current manufacturing facilities. Using the here presented method for semi-batch cell culture and subsequent virus culture, d-antigen yields per run can be doubled.

Concluding, the use of increased cell densities can result in more efficient use of bioreactor capacity, and may so reduce the vaccine cost per dose.

## Figures and Tables

**Table 1:** Cell culture characteristics of different Vero cell cultivation methods.

Cultivation method	Inoculation cell density (×10^6^ cells mL^−1^)	Cell density at time of infection (×10^6^ cells mL^−1^)	Culture time (days)	Growth rate at virus infection (h^−1^)
Batch (*n* = 5)	0.10 ± 0.04	1.02 ± 0.07	4	0.022
Semi-batch (*n* = 3)	0.14 ± 0.01	1.75 ± 0.32	6	0.006
Perfusion (*n* = 3)	0.08 ± 0.01	2.72 ± 0.10	7	0.005
Recirculation (*n* = 3)	0.64^a^ ± 0.11	5.01 ± 0.40	4^a^	0.0004

a Requires a larger pre-culture volume.

**Table 2:** Virus titers and d-antigen concentrations observed using different Vero cell culture methods before virus replication.

Cultivation method during cell culture	Poliovirus type 1		Poliovirus type 2		Poliovirus type 3	
	Virus titer (10 LOG TCID_50_ mL^−1^)	d-antigen (DU mL^−1^)	Virus titer (10 LOG TCID_50_ mL^−1^)	d-antigen (DU mL^−1^)	Virus titer (10 LOG TCID_50_ mL^−1^)	d-antigen (DU mL^−1^)
Batch	8.7	175	8.8	44	8.8 ± 0.8 (*n* = 3)	78 ± 6 (*n* = 3)
Semi-batch	8.8	356	8.7	72	7.9	137
Perfusion	9.3	320	8.3	65	8.6	115
Recirculation	9.5	508	8.8	115	8.9	185
